# Nurse-based case management for aged patients with myocardial infarction: study protocol of a randomized controlled trial

**DOI:** 10.1186/1471-2318-10-29

**Published:** 2010-05-27

**Authors:** Inge Kirchberger, Christa Meisinger, Hildegard Seidl, Rupert Wende, Bernhard Kuch, Rolf Holle

**Affiliations:** 1 Institute of Epidemiology, Helmholtz Zentrum München, German Research Center for Environmental Health, Neuherberg, Germany; 2 MONICA/KORA Myocardial Infarction Registry, Central Hospital of Augsburg, Augsburg, Germany; 3 Institute of Health Economics and Health Care Management, Helmholtz Zentrum München, German Research Center for Environmental Health, Neuherberg, Germany; 4 Department of Internal Medicine I - Cardiology, Central Hospital of Augsburg, Augsburg, Germany

## Abstract

**Background:**

Aged patients with coronary heart disease (CHD) have a high prevalence of co-morbidity associated with poor quality of life, high health care costs, and increased risk for adverse outcomes. These patients are often lacking an optimal home care which may result in subsequent readmissions. However, a specific case management programme for elderly patients with myocardial infarction (MI) is not yet available. The objective of this trial is to examine the effectiveness of a nurse-based case management in patients aged 65 years and older discharged after treatment of an acute MI in hospital. The programme is expected to influence patient readmission, mortality and quality of life, and thus to reduce health care costs compared with usual care. In this paper the study protocol is described.

**Methods/design:**

The KORINNA (Koronarinfarkt Nachbehandlung im Alter) study is designed as a single-center randomized two-armed parallel group trial. KORINNA is conducted in the framework of KORA (Cooperative Health Research in the Region of Augsburg). Patients assigned to the intervention group receive a nurse-based follow-up for one year including home visits and telephone calls. Key elements of the intervention are to detect problems or risks, to give advice regarding a broad range of aspects of disease management and to refer to the general practitioner, if necessary. The control group receives usual care. Twelve months after the index hospitalization all patients are re-assessed. The study has started in September 2008. According to sample size estimation a total number of 338 patients will be recruited. The primary endpoint of the study is time to first readmission to hospital or out of hospital death. Secondary endpoints are functional status, participation, quality of life, compliance, and cost-effectiveness of the intervention. For the economic evaluation cost data is retrospectively assessed by the patients. The incremental cost-effectiveness ratio (ICER) will be calculated.

**Discussion:**

The KORINNA study will contribute to the evidence regarding the effectiveness of case management programmes in aged people with MI. The results can be an important basis for clinicians, administrators and health policy makers to decide on the provision of high-quality care to older patients with CHD.

**Trial registration:**

ISRCTN02893746

## Background

### Coronary heart disease (CHD) in aging populations

The aging of the population and the increasing prevalence of chronic diseases imply great challenges to the health systems of developed countries. CHD is the leading cause of mortality and morbidity in the industrialized world. The treatment of acute myocardial infarction (MI) has improved dramatically over the last 10 to 20 years and nowadays, aged patients with an acute MI are receiving treatment that had been limited to mainly younger patients about a decade ago [1]. Subsequently, the number of survivors with MI will increase in the next decades.

Patients with CHD, in particular aged patients, have a high prevalence of co-morbidity associated with poor quality of life, physical disability, high health care costs, multiple medications, and increased risk for adverse outcomes. Common co-morbidities among patients with CHD are diabetes, chronic heart failure (CHF), chronic obstructive pulmonary disease (COPD), and depression [1-3]. CHD is the most common reported cause of chronic heart failure in all age-groups [4]. A number of studies also found that more aged than young patients develop CHF following an acute MI: 47% of those aged greater than 75 years versus 23% aged < 75 years [5]. In addition, diabetes is not only a co-morbidity of CHD, but is also an independent risk factor for the development of CHF particularly in the aged [6]. CHF is the most common cause of hospital admission in the aged [7], and more aged than younger patients with CHF are discharged to long-term care and this number is increasing [8]. Thus, as a rule, aged patients do not present with an isolated medical problem, but have multiple co-morbid diseases [9]. Consequently, these patients receive a number of medications [10].

Due to changes in the German health care system, in particular the establishment of the diagnosis related groups (DRG) system, older patients with complex health care needs are nowadays discharged earlier from hospital than a few years ago [11]. For many of these patients an optimal care at home is not guaranteed, resulting in subsequent readmissions or final admission to a nursing-home. Medication compliance and lack of medication-related knowledge are serious problems in aged patients, and in higher age-groups there is also often a lack of social support or knowledge to seek medical support promptly, when specific symptoms appear [12]. Thus, optimizing care for this population has a high priority.

Many hospital admissions among aged patients with CHD can be attributed to behavioural and social factors rather than to deteriorating cardiac function or an intercurrent cardiac event [13]. Thus, aged multimorbid patients at risk of poor outcomes might benefit from intensive home follow-up. A nurse-led home-based intervention program including patient education and counselling might improve medication compliance, medication-related knowledge and thus processes of care, coronary risk factor profiles, functional status, and quality of life. As a consequence, this might reduce mortality. In addition, such a program is expected to reduce health care resource use in patients with CHD and consequently total health care expenditures.

### Case management programmes for patients with CHD

A number of intervention trials investigated whether a nurse-based case management may influence patient readmission and other outcomes in CHD [14,15]. In a meta-analysis investigating the effectiveness of secondary prevention programmes with and without exercise components 63 randomized trials were included [14]. Almost all included studies were conducted in patients with CHD younger than 70 years. It could be shown that secondary prevention programmes positively affect process of care, survival, and functional status or quality of life of patients with CHD independent of the applied programme.

In another review McAlister et al. examined whether case management programmes for patients with established CHD improve the process of care and reduce mortality [15]. In total 12 randomized trials also including mainly patients younger than 70 years could be identified by the investigators. It could be demonstrated that comprehensive case management programmes have a positive effect on processes of care in patients with CHD: there was a significant reduction in admissions to hospital and an improvement in quality of life. However, these randomized clinical trials failed to document any survival benefit or reduction in recurrent MI.

Although the results of both studies, the meta-analysis and the review, are promising, these findings can not be generalized to higher age-groups. Very few studies reported on case management programmes in people older than 65 years. The studies by Naylor et al [16,17] examined a nurse-centered discharge planning and home follow-up intervention in patients older than 65 years with a broad variation of diagnoses and interventions. Their study on older people with CHF (mean age 76 years) demonstrated that the intervention significantly increased the length of time between hospital discharge and readmission or death, reduced total number of rehospitalizations, and decreased healthcare costs [17].

Regarding cost-effectiveness of the case management programmes it is notable that only seven of the 63 trials included in the meta-analysis by Clark et al. [14] described the costs of the intervention. Two reported that their intervention was cost saving but only one has performed formal cost-effectiveness analyses. From the twelve studies included in the review by McAlister et al. [15], three described cost data and two reported that their intervention was cost saving, but none performed formal cost-effectiveness analyses. Based on the observed lack of formal economic evaluations in these studies and the conflicting results, the need to systematically integrate economic evaluations into such studies was highlighted by the authors [14,15].

Overall, the review of the literature showed that studies on case management programmes mostly included participants with CHD younger than 70 years [14] or included persons hospitalized with one of several common medical and surgical reasons [18,19]. So far, no intervention trial focused on a specific case management programme for the group of aged patients after MI. Case management focuses on delivering personalized services to patients to improve their care. Case management after hospital discharge could play a central role for the prevention of re-infarction or readmission for other reasons among aged patients with an acute MI.

### Objective of the study

The primary objective of the study is to assess whether a case management intervention by trained nurses can reduce time to first readmission or out-of-hospital death in aged patients with MI. Secondary objectives are the estimation of the cost-utility ratio of this case management intervention and to compare case management vs. routine care with respect to secondary out-comes such as functional capacity, participation, quality of life, and compliance with medication. For patients with specific comorbidity, additional outcome criteria will be compared between the two groups, e.g. adjustment of blood glucose values and hemoglobin-A_1c _in patients with type 2 diabetes, and maintenance of body weight in patients with CHF.

The aim of this paper is to describe the study protocol of the KORINNA (Koronarinfarkt-Nachbehandlung im Alter) study. Patient recruitment in the KORINNA study has started by September 2008. This detailed description of the study background and protocol is published as a reference for forthcoming papers on the study results.

## Methods/Design

### Study design

**Figure 1:**
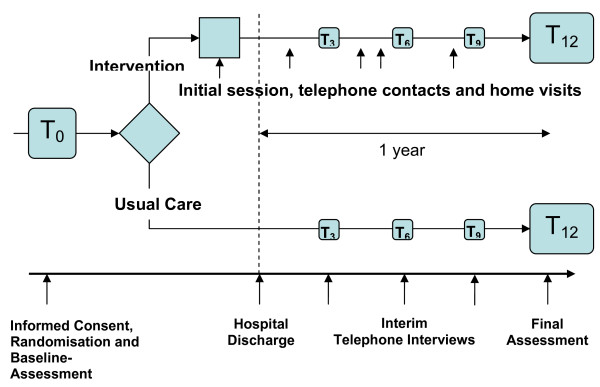
**Study design**.

The study is designed as a single-centre randomized two-armed parallel group trial. The study is carried out within the framework of KORA (Cooperative Health Research in the Region of Augsburg). All patients receive a baseline assessment after giving their informed consent. The baseline assessment is carried out by the study physician and a study nurse. Those who are subsequently randomized to the intervention group receive the intervention starting with the initial session shortly before discharge (see Figure 1). All patients are contacted by phone after 3, 6, and 9 months in order to document a core set of relevant outcome measures. The duration of the intervention phase is one year. After one year a final investigation takes place in the hospital or at home.

### Patient selection

#### Inclusion criteria

In the KORINNA study all patients of the age group 75 years and older with a first or recurrent MI during the planned recruitment period who are treated in the Augsburg Hospital should be included. The Augsburg Hospital is the largest hospital in the region of Augsburg - a city with 300,000 inhabitants in the south of Germany - offering a coronary care unit as well as coronary angiography and angioplasty facilities 24 hours a day. All myocardial infarctions according to ESC and ACC criteria will be included [20].

However, an unexpected low number of patients of the age group 75 years and older became apparent during the first year. As a consequence, the study protocol was modified and the minimum age of participants was set to 65 years. The recruitment phase needs to be expanded accordingly. This modification was discussed with the studies' Advisory Board.

#### Exclusion criteria

Patients who already live in institutionalized care are excluded. In addition, patients who already plan to move into institutional care or outside the study region within the next months are excluded as well. Furthermore, patients with severe comorbidity (e.g. terminal cancer) which makes rehospitalization within the next months necessary or is associated with a life expectancy of less than one year, and patients who are not able to communicate in German language are excluded. Finally, patients who are unable or unwilling to give written informed consent (e.g. patients with dementia) cannot be included in the study. The number of patients not included in the study as well as reasons for exclusion will be documented.

The study protocol and its amendment were approved by the Ethics Committee at the Bavarian Chamber of Physicians (Date of approval: 11.11.2008, Reference number: 08064). Furthermore, the study is conducted in accordance to German privacy law and in compliance with the Helsinki Declaration.

### Randomization

All patients meeting the inclusion criteria and giving informed consent are randomly assigned to the intervention or control group. The allocation ratio is 1:1. To ensure the concealment of the allocation, randomization is provided per telephone call to the biostatistical center at the Helmholtz Zentrum München, German Research Center for Environmental Health, where a randomization list is kept. A minimization procedure is used which tries to achieve balanced treatment groups with respect to gender, age (< 70 vs. 70-79 vs. 80+), and number of comorbidities (diabetes and CHF).

### Intervention

The intervention consists of a nurse-led individualized home-follow up programme with a duration of one year. The control group receives usual care. Patients can use or apply for all available services in the area.

#### Initial session before discharge

The intervention programme starts with an initial information session (approximate duration 1 hour) which takes place one or two days before hospital discharge (see Figure 1). In this session the patient is first of all provided with information about the disease and comorbidities, about medication and with behavioural recommendations (nutrition, physical activity, smoking etc.). Information is given orally and in written form (so-called "heart book"). If possible, close relatives of the patient may participate in the discharge session. A first home visit is arranged, if accepted by the patient, otherwise an appointment for a telephone call is made.

#### Home visits and telephone calls

Home visits (0 to 4) and telephone calls (at least every 3 months) are carried out according to patient need and patient risk level. At the first home visit the specific problems of the patient are identified and documented. An individual plan for each patient is set up. The risk level is assessed by the study nurse during the first home visit based on compliance, the social network, and the comorbidities. The higher the risk level the more contacts (telephone and home visits) are arranged by the study nurse. The activities are planned in agreement with the patient in order to improve compliance.

The first home visit is scheduled to take place 7 to 14 days after discharge. If the patient stays in a rehabilitation hospital immediately after discharge from the Augsburg hospital, the first home visit will be postponed accordingly. At the home visits patients are instructed how the prescribed drugs have to be taken and what would happen in the case of non-compliance with medication. Furthermore, to patients with diabetes advice is given regarding nutrition and physical activity, and patients with CHF are encouraged to regular weight control. During the visits, measurements of blood glucose, blood pressure, and weight are performed. The duration of the visit should be between 60 and 90 minutes. The key elements of both the home visits and the telephone calls are: to detect problems (e.g. regarding intake of medication) or risks, to give advice regarding a broad range of aspects of disease management (e.g. health behaviour) and to refer to the general practitioner, if necessary. The content and structure of home visits and telephone calls are comparable, and primarily differ regarding location and type of contact between the patient and nurse, and the possibility to measure blood glucose, blood pressure, and weight.

#### Training

In August 2008, a training of the three study nurses and the study physician was performed lasting several days. The following topics and diseases were intensively discussed with the study nurses: CHD, MI, angina pectoris, heart failure, treatment of MI, risk factors of cardiovascular diseases, action of particular drugs, diabetes mellitus, psychological factors and MI. Furthermore, the structure and the contents of the "Heart book" handed out to the patients in the intervention group at discharge were discussed.

Regarding the study implementation, both study physician and study nurses received a training on patient recruitment. The study physician was instructed on patient information and obtaining informed consent. Moreover, the procedure of the clinical examination and personal interview was trained.

Finally, study nurses were introduced in the performance of personal interviews in the hospital, of home visits, and of telephone interviews, the use of the intervention modules, the data extraction from patients' records, documentation, and data entry.

### Baseline and outcome assessments

The primary endpoint of readmission is measured as time between initial hospital discharge and first unplanned readmission to hospital (at least for 24 hours). Out of hospital death from any cause is counted as an event.

The instruments used to assess secondary outcomes are shown in Table 1. Baseline data on all recruited patients (intervention and control group) are collected at the index hospitalization, usually one or two days before the patient will be discharged. Data on sociodemographic and health status characteristics, functional status, participation, and psychosocial status are collected by the study physician and a trained study nurse. After discharge, standardized non-interventional telephone interviews with the patients in both groups are planned at 3, 6, and 9 and 12 months after index hospital discharge identifying patient's readmissions, acute care visits to physicians, clinics, and ambulatory departments. Data on quality of life is also collected during these interviews. In the intervention group additional telephone calls as well as home visits take place as part of the intervention. Additional information is documented on these opportunities in order to monitor process quality.

**Table 1:** Outcome measures

Category	Instrument	Reported by	T_0_	T_3_	T_6_	T_9_	T_12_
Anamnesis	Baseline questionnaire	Physician	•				
Anamnesis	Geriatric Assessment [26]	Physician	•				
Quality of life	EQ-5D [24]	Patient	•	•	•	•	•
Quality of life	WHO-5 Well Being Index (WHO-5) [27]	Patient	•				•
Cognitive functions	Mini Mental State Exam (MMSE) [28]	Physician	•				•
ADL	Barthel Index [29]	Physician	•				•
IADL	Instrumental Activities of Daily Living Scale (IADL) [30]	Physician	•				•
Functioning	Health Assessment Questionnaire (HAQ-DI) [31]	Patient	•				•
Functioning	Hand grip strength measurement [32]	Physician	•				•
Mobility	Timed up and go (TUG) [33]	Physician	•				•
Nutrition	Seniors in the Community Risk Evaluation for Eating and Nutrition (SCREEN II, Version II) [34]	Patient	•				•
Social Support	Fragebogen zur sozialen Unterstützung (F-sozU) [35]	Patient	•				•
Depression	Geriatric depression scale (GDS) [36]	Patient	•				•
Resource use	Questionnaire	Patient	•	•	•	•	•

Twelve months after the index hospitalization all patients who are still alive are invited for a final assessment in the hospital or, if necessary, at home. This examination includes a personal interview with an assessment of functional capacity, mental status and quality of life, adherence to medication, health care utilization, and the measurement of blood pressure and body weight. The study physician and nurse who are involved in the final assessment are not aware whether the respective patient was assigned to the intervention- or control group.

Data on resource utilization as well as on quality of life are collected at baseline, at interim telephone contacts, and during the final assessment.

### Economic evaluation

Data for the economic analysis is collected via patients and from hospital records. Table 2 summarizes the data collection for the economic evaluation. As valid information on the resource use can only be retrospectively gathered for a short previous time period the assessment of resource utilization is conducted quarterly.

**Table 2:** Overview on the cost categories, the measurement of the resource use and its valuation

Cost category	Resource use	Valuation
Outpatient care	Number of consultations	€ per consultation
Prescribed drugs	Central pharmaceutical number, ATC code, dose rate	Pharmacy retail price
Inpatient care	Length of stay and number of days on the intensive care unit, if applicable DRG	Daily hospital rate, if applicable DRG
Rehabilitation (excluding primary rehab)	Length of stay	Daily rehabilitation rate
Remedies (physiotherapy, massage)	Number of visits	Average rates
Ambulatory care	Days per week and performed activities (or hours per day)	Average rates for activities (or hours)
Home help	Hours per week	Average labor costs for a home help
Informal care	Care level declared by the long term care insurance	Max amount paid by the long term care insurance if the patient does not make use of formal care

Within the last years, standardized instruments measuring the resource use were developed such as the RAI (Resident Assessment Instrument) [21] or the RUD (Resource utilization of dementia) [22]. Based on these questionnaires and on our experiences we developed a questionnaire for the patients that can be used in personal and telephone interviews.

The valuation of the resource use will be based on the valuation rates calculated by Krauth et al. [23]. As the valuation rates are based on data for the year 2000 the calculations will be up-dated.

Especially in the economic evaluation of interventions with the elderly informal care plays an important role. However, as the assessment is very time consuming and the interview time should be minimized we refrained from assessing informal care time. To estimate the cost of informal care we use the information on a care level declared by the long term care insurance. If the patient is allowed to receive nursing care and does not receive formal care, it is assumed that the patient has an informal caregiver. In that case, the maximum amount for care at home paid by the long term care insurance will be used as cost for informal care.

Cost for the intervention excluding the costs caused by the study will be considered. Cost components will include labor costs, travel expenses, telephone costs etc.

In order to calculate quality-adjusted life years (QALYs), the EQ-5D questionnaire [24] is applied at baseline, at interim telephone contacts, and during the final assessment. Additionally, the Visual Analogue Scale (VAS) [24] is applied at baseline and final assessment.

It is planned to perform a cost-utility-analysis from the societal perspective. This means that incremental costs of the intervention (vs. control) are set in relation to the number of QALYs gained. The resulting incremental cost-effectiveness ratio (ICER) is an international standard for reporting results of economical evaluation studies. In addition, we will calculate additional costs per life year saved and per year without event. For the estimated ICER value a 95% confidence interval will be calculated based on bootstrap methods.

### Analysis

The statistical analysis for the primary endpoint will focus on the null hypothesis that there is no difference between intervention and control with respect to the distribution of time to un-planned readmission (including out-of-hospital death). This hypothesis will be statistically tested using a Cox proportional hazards model including age, gender, and comorbidity as covariates.

Statistical analyses for secondary outcomes will be based on (generalized) linear models depending on the type and distribution of the outcome variable. Since these will be multiple tests for which control of the overall error level cannot be achieved, these will be regarded as exploratory analyses. All analyses will be done according to the intent-to-treat approach.

### Sample size

Based on a comparable study by Young and colleagues [25] with patients 70 years of age we expect an event rate (readmission or out-of-hospital death) of 40% in the control group. If the study is designed to have 80% power or more to detect an improved rate of 25% in the intervention group (i.e. Δ = 0.15) at a two-sided type I error level of 5%, at least 152 patients per group will be needed. We expect that the drop-out rate does not exceed 10% during the 1-year follow-up period. In order to allow for loss to follow-up (patient withdrawing consent or moving away from study region), it is planned to recruit a total of 338 patients for the trial.

## Discussion

This paper describes the study design of the KORINNA study, an innovative randomized controlled trial evaluating the effectiveness of a nurse-based case management programme for old-aged patients with MI. The KORINNA study is unique in a twofold way.

First, the nurse-led case management is tailored to the specific needs of old-aged people with MI. The interventions specifically address common challenges and risks associated with the MI management in elderly people such as multi-morbidity and compliance with medication. Further, the programme is flexible regarding frequency and location of the intervention. Thus, when deciding on the number of interventions necessary or whether a home-visit or telephone call is more appropriate, the nurse can take the individual health and living condition of a patient into account. Our experiences gathered from approximately 100 patients who have already received interventions within the study, confirm the need for a highly individualized intervention programme. Until now, a broad range of health states and impairments among the study participants could be observed which necessitates a variable intervention intensity.

Second, a particular strength of this study relates to the planned economic evaluation. In view of scarce financial resources within the German health system it is essential that innovative models of care not only demonstrate positive effects on clinical and patient-related outcomes but also cost-effectiveness. Since cost savings have already been shown in comparable studies in other countries, the economic results of this study are highly anticipated.

A possible limitation of the study design refers to the relatively short follow-up interval of one year. It is still unclear whether the expected positive intervention effects on readmission to hospital or death can already be observed within this time interval. Hence, a prolongation of the study with a follow-up interval of 3 years is already taken into consideration. A further limitation of the study is its single center design.

So far, we have experienced an unexpected challenge regarding the number of eligible patients aged 75 years and older. Some months after starting recruitment it became apparent that the planned sample size cannot be reached within the expected time frame. We discussed this problem with the members of the studies' advisory board and decided to decrease the minimum age of study participants from 75 years to 65 years. As a consequence of the initially low number of patients who could be included in the study, the recruitment phase will be extended by several months. In addition, the number of patients who could not be included due to severe concomitant diseases or dementia (20%), or since they were already nursing home residents (10%), was higher than expected. On the other hand, the number of study patients who were lost to follow-up within the one-year treatment course currently is 7% and therefore even lower than initially expected. In addition, we have noticed that the home visits as well as telephone calls are very well accepted by the patients. These findings indicate a good acceptance and feasibility of the study.

In summary, the KORINNA study is expected to contribute to the evidence regarding the effectiveness of nurse-based case management programmes for aged patients with MI. The results can be an important basis for clinicians, administrators and health policy makers to decide on the provision of high-quality care to older patients with MI.

## Competing interests

The authors declare that they have no competing interests.

## Authors' contributions

CM, BK and RH initiated the study and raised the research grant. CM and BK are principal clinical investigators, RH is principal statistical investigator. CM and BK developed the case-management programme. CM, RH, BK, RW, HS contributed to conception and design of the study. HS is responsible for the economic evaluation and for study monitoring. RW is study physician and responsible for patient recruitment and clinical examination/assessment. IK is in charge of the local coordination of the study and wrote the manuscript. All authors critically revised and approved the final manuscript.

## Pre-publication history

The pre-publication history for this paper can be accessed here:

http://www.biomedcentral.com/1471-2318/10/29/prepub
